# Effect of LysM+ macrophage depletion on lung pathology in mice with chronic bronchitis

**DOI:** 10.14814/phy2.13677

**Published:** 2018-04-18

**Authors:** Yogesh Saini, Brandon W. Lewis, Dongfang Yu, Hong Dang, Alessandra Livraghi‐Butrico, Fabio Del Piero, Wanda K. O'Neal, Richard C. Boucher

**Affiliations:** ^1^ Department of Comparative Biomedical Sciences School of Veterinary Medicine Louisiana State University Baton Rouge Louisiana; ^2^ Marsico Lung Institute/Cystic Fibrosis Center School of Medicine University of North Carolina at Chapel Hill Chapel Hill North Carolina; ^3^ Department of Pathobiological Sciences School of Veterinary Medicine Louisiana State University Baton Rouge Louisiana

**Keywords:** Airway inflammation, airway mucus obstruction, alveolar macrophages, MΦ depletion, *Scnn1b* transgenic mice

## Abstract

Macrophages (MΦ) are key sentinels of respiratory exposure to inhaled environmental stimuli. In normal “healthy” tissues, MΦ are believed to be a dormant cell type that, upon exposure to stress‐causing stimuli, may get activated to exhibit pro‐ or anti‐inflammatory roles. To test whether stress present in chronic bronchitic (CB) airways triggers MΦ to manifest protective or detrimental responses, the DTA+ (LysM‐regulated Diphtheria Toxin A expressing) strain with partial MΦ‐deficiency was crossed with the *Scnn1b*‐Tg mouse model of CB and the progenies were studied at 4–5 weeks of age. Compared with DTA− littermates, the DTA+ mice had ~50% reduction in bronchoalveolar lavage (BAL) MΦ, and the recovered MΦ were immature, phenotypically distinct, and functionally defective. DTA+/*Scnn1b*‐Tg mice exhibited a similar depletion of LysM+ MΦ offset by a significant increase in LysM‐ MΦ in the BAL. In DTA+/*Scnn1b*‐Tg mice, lung disease was more severe than in DTA−/*Scnn1b*‐Tg littermates, as indicated by an increased incidence of mucus plugging, mucous cells, airway inflammation, higher levels of cytokines/chemokines (KC, TNF‐*α*, MIP‐2, M‐CSF, IL‐5, and IL‐17), and worsened alveolar airspace enlargement. DTA+/*Scnn1b*‐Tg mice exhibited increased occurrence of lymphoid nodules, which was concomitant with elevated levels of immunoglobulins in BAL. Collectively, these data indicate that numerical deficiency of MΦ in stressed airspaces is responded via compensatory increase in the recruitment of immature MΦ and altered non‐MΦ effector cell‐centered responses, for example, mucus production and adaptive immune defense. Overall, these data identify dynamic roles of MΦ in moderating, rather than exacerbating, the severity of lung disease in a model of CB.

## Introduction

A balanced composition of immune cells in the respiratory tract is critical to normal host defense and responses to disease. Numerical or functional deficiencies of macrophage (MΦ) populations in the airspaces (airway and alveolar) of normal lungs is predicted to modulate host defense responses associated with pathogen invasion, environmental toxicants exposure, and intrinsic or genetic defects. In disease, activation of MΦ may serve either to: reduce disease severity, via persistent anti‐inflammatory effects and/or anti‐microbial activities (Burnett et al. 2004); or worsen the disease phenotype, via persistent but inappropriate inflammatory responses (Byrne et al. 2015). Therefore, a detailed understanding of complex interactions between airspace MΦ and disease severity in chronic airway disease is challenging to predict and requires direct testing.

The initiating events in airspace diseases include the exposure to extrinsic biotic and abiotic agents and/or intrinsic defects in the normal functioning of epithelial and immune cells. Mucus hyperconcentration, a common feature in multiple types of chronic bronchitis (CB), may act as a trigger to modulate MΦ responses and/or recruit MΦ. The epithelial sodium channel beta subunit (*Scnn1b)* transgenic mouse is a model of CB that exhibits airway surface liquid (ASL) dehydration‐induced mucus accumulation/adhesion characterized by persistent mucous cell metaplasia, neutrophilic inflammation, and intermittent early postnatal infection (Mall et al. 2004, 2008). Resident MΦ are key sentinel cells on airway and alveolar surfaces that play a critical role in the initiation, progression, and resolution of pulmonary inflammatory responses. Pulmonary MΦ in *Scnn1b*‐Tg mice exhibit activation features beginning at birth, suggesting their role as an “early responder” cell‐type in the development of CB (Saini et al. 2014). Neonatal MΦ‐depletion worsens the neonatal *Scnn1b*‐Tg lung phenotype by the spread of the highly penetrant bacterial infection to the alveolar spaces and increasing neutrophilic inflammation, demonstrating that MΦ are critical to the containment and eradication of early neonatal bacterial infections (Saini et al. 2015). However, the effect of MΦ depletion on the phenotype of 4‐ to 5‐week‐old *Scnn1b*‐Tg mice, which have persistent neutrophilic and eosinophilic inflammation but only intermittent bacterial infection, is unknown.

To elucidate the roles of MΦ in the pathogenesis of CB, *Scnn1b*‐Tg mouse with MΦ‐depletion was generated by crossing *Scnn1b*‐Tg mice with DTA+ (expresses Diphtheria Toxin‐A in MΦ) mice and the progenies were analyzed at the age of 4–5 weeks. We hypothesized that pulmonary MΦ sense the hyper‐concentrated mucus and/or non‐infectious inflammatory stimuli trapped within static mucus and respond through increased MΦ activation to modulate epithelial and immune cell responses. We further hypothesized that MФ depletion would add a new component to the adult *Scnn1b*‐Tg phenotype, that is, worsening of the airway CB phenotype, due to the absence of macrophage‐specific anti‐inflammatory activities, and due to inefficient bacterial clearance. To test these hypotheses, the effects of LysM‐Cre regulated DTA expression on MΦ numbers and function in WT (without *Scnn1b* transgene) and *Scnn1b*‐Tg (with *Scnn1b* transgene) mice, the incidence of bacterial infection in *Scnn1b*‐Tg mice, and the effects of numerical/functional deficiencies of MΦ populations on airway and alveolar pathology of *Scnn1b*‐Tg mice were investigated.

## Materials and Methods

### Transgenic mice and animal husbandry

MΦ‐specific promoter (LysM)‐regulated Cre‐recombinase (Cre) expressing line [(B6.129P2‐*Lyz2* ^*tm1(cre)Ifo*^/J) on C57BL/6N background], ROSA promoter‐regulated mTom^flox/flox^/mEGFP expressing line [(B6.129(Cg)Gt(ROSA)26Sortm4(ACTB‐tdTomato,‐EGFP)Luo/J) on C57BL/6J background], and ROSA promoter‐regulated (neo/STOP cassette)^flox/flox^/DTA expressing line [(B6.129P2*Gt(ROSA) 26Sor* ^*tm1(DTA)Lky*^/J) on C57BL/6J background] were procured from Jackson Laboratory (Bar Harbor, ME). At the time of procurement, LysM‐Cre mice were on mixed C57BL/6N and C57BL/6J background whereas mTom/mEGFP reporter mice and DTA‐floxed mice were on C57BL/6J background. *Scnn1b‐*Tg mice used in the study were C57BL/6N congenic. WT mice refer to mice without *Scnn1b* transgene. All mice used in the study were maintained in hot‐washed, individually ventilated micro‐isolator cages on a 12‐hour dark/light cycle and were fed regular diet and water ad libitum. Studies were performed on 4‐ to 5‐week‐old mice from four different transgenic lines. All animal procedures were performed using animal use protocol (Protocol ID 10.226; IACUC # 23999) approved by the Institutional Animal Care and Use Committee of UNC‐CH.

### Generation of MΦ depleted mouse models and PCR genotyping

Various mouse models of MΦ labeling with mEGFP, MΦ depletion, and MΦ‐depleted *Scnn1b*‐Tg mice were generated (Fig. S1). PCR genotyping was performed as previously reported (Saini et al. 2015). MΦ‐depleted mouse with genotypic configuration, LysM‐Cre^+/−^/DTA^+/−^ will be referred to as DTA+. The control mice with genotypic configuration, LysM‐Cre^+/−^/DTA^−/−^ will be referred to as DTA−.

### Bronchoalveolar lavage collection and lung tissue processing

Bronchoalveolar lavage (BAL) was harvested for differential cell counts, fluorescent microscopic analyses, and microaerophilic bacterial burden estimation as previously described (Livraghi et al. 2009; Saini et al. 2015).

### Flow cytometry

BAL cells were collected as described previously. Cells were fixed in a 1‐step fix/lyse solution (eBiosciences, CA), washed twice in PBS, and the pellets were suspended in staining buffer. BAL cells were analyzed for the mEGFP and mTom fluorescence with Dako CyAn (Beckman Coulter, Inc., CA). Flow cytometric data were analyzed using Summit software Version 4.3 (Dako, CA).

### Apoptosis assays

BAL MΦ from WT and LysM‐Cre^+/−^/DTA^+/−^ (DTA+: MΦ‐depleted) mice were plated in 96‐well cell culture plates in MΦ culture media (RPMI 1640 medium (Life Technologies, VA) supplemented with 10% fetal bovine serum and 100 *μ*g/mL streptomycin/penicillin (Sigma, MO). Apoptotic MΦ was labeled with a non‐fluorescent substrate (four amino acid [DEVD] peptide quenched fluorescent dye) of caspase3/7 (CellEvent Caspase‐3/7 Green Detection Reagent, Life Technologies, VA). The cleavage of this peptide by activated caspase 3/7 converts non‐fluorescent substrate to green fluorescent DNA labeling dye. The assays were performed according to manufacturer's protocol (Life Technologies, VA). Briefly, after 1 hour of adherence, MΦ culture media with the CellEvent Caspase‐3/7 Green Detection Reagent (Life Technologies, VA) was added and plates were incubated at 37°C for 1 hour. After incubation, media was removed and cells were fixed with 4% paraformaldehyde followed by DNA labeling with 5 *μ*g/mL solution of Hoechst 33,342 solution in PBS (BD Biosciences, CA). Apoptotic cells with green fluorescent‐labeled DNA were imaged with a Leica DMIRB inverted fluorescence microscope (UNC Michael Hooker Microscopy Facility).

### Histopathological slide preparation

Ten percent neutral buffered formalin‐fixed unlavaged left lung lobes were paraffin embedded. The transverse sections were made at the level of proximal intrapulmonary main axial airway near the hilus and subsequently, at a distance of 2 mm towards the base of the lung. 4–6 *μ*m thick sections were mounted on glass slides and stained with hematoxylin and eosin (H&E) for lung morphological assessments and Alcian Blue/Periodic Acid‐Schiff (AB‐PAS) for mucopolysaccharide contents in airway lumen and airway epithelium.

### Semiquantitative histopathological assessment

A previously reported semiquantitative grading approach was used to score airway inflammation, airway obstruction, mucous cell abundance, and airspace enlargement (graded on a 0–3 scale) (Livraghi et al. 2009). Alveolar space consolidation was scored as previously reported (Saini et al. 2015).

### Morphometric analysis of alveolar topology

A morphometric method was used to provide a quantitative measure of the histological observations in alveolar airspace topology among genotypes. Briefly, unlavaged lung (left lobe) sections were stained with H&E. Photomicrographs of the parenchyma were taken using the Micropublisher camera (using Q imaging and Q capture software) and assembled on Leica DMIRB inverted fluorescence/DIC microscope. Twelve images were captured at fixed magnification from H&E stained lung sections. Images were captured from regions that did not contain large airways or vessels and were selected from regions not affected visually by consolidation or containing lymphoid nodules or visually obvious inflammation. An investigator blinded to genotype counted the visible airspaces defined by alveolar walls in all 12 sections from each animal. The total number of alveoli (Airway Space Enlargement Index [ASEI]) was calculated in all the 12 photographs and data was analyzed (Fig. S3). While this method cannot address the true size of alveolar airspaces, or distinguish emphysema from air trapping, it was sensitive to detect alterations in alveolar airspace across the four genotypes. The effects of genotype and MΦ depletion on ASEI values were fitted through linear regression with standard least square model, together with experimental factors, such as batch and tissue slice depth using JMP version 8.0.2 (SAS Institute Inc., Cary, NC). The effect of genotype, MΦ depletion status, and potential interaction between them were estimated using multi‐variant ANOVA and Tukey HSD post hoc tests.

### BAL cytokines and immunoglobulin isotype analyses

Mouse KC, MIP‐2, LIX, TNF*α*, MIP‐1*α*, MCP‐1, M‐CSF, IL‐10, IL‐17, IL‐4, IL‐5, IL‐6, IP‐10, Eotaxin, and IL1*β* levels were assayed in BAL supernatant (after centrifuging at 10,000*g* for 10 minutes) using a Luminex‐XMAP based assay (MCYTOMAG‐70K), according to the manufacturer instructions (EMD Millipore Corporation, Billerica, MA). To estimate BAL concentration of six major immunoglobulin isotypes, i.e., IgA, IgM, IgG1, IgG2a, IgG2b, and IgG3, BAL supernatant was analyzed using a Luminex‐XMAP based assay (MGAMMAG‐300K), according to the manufacturer instructions (EMD Millipore Corporation, Billerica, MA).

### Statistical analyses

Statistical analyses were performed using GraphPad Prism 6.0 (La Jolla, CA). One‐way Analysis of Variance (ANOVA) followed by Tukey's post hoc test for multiple comparisons was used to determine significant differences among groups. All data were expressed as mean ± standard error of the mean (SEM). *P* value <0.05 was considered statistically significant.

## Results

### LysM promoter activity labels the entire pulmonary MΦ population

**Figure 1:**
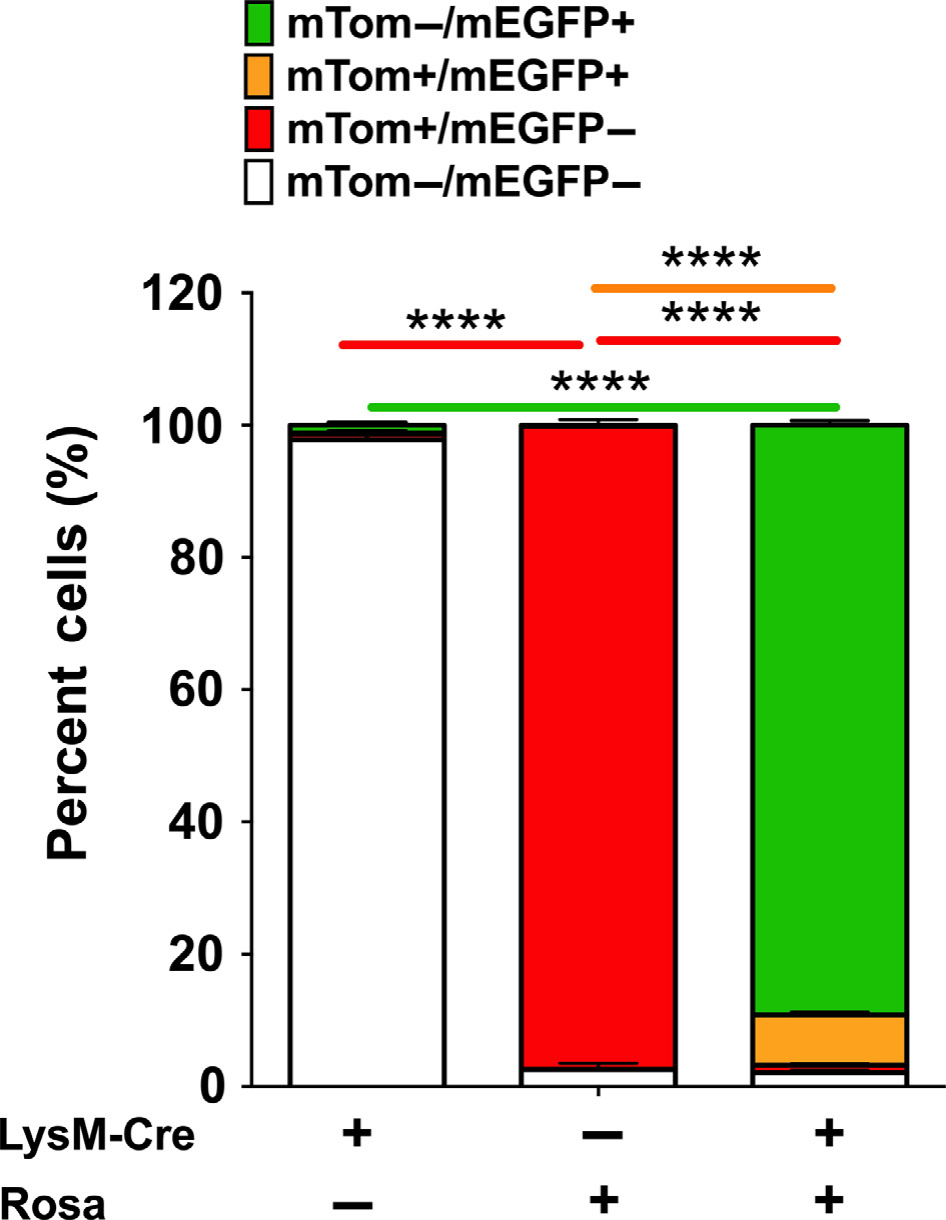
Lysozyme M promoter exhibits robust activity in the older age. Flow cytometry analysis was performed on BAL cells harvested from LysM‐Cre+\ROSA‐mTom/MEGFP‐ (Left bar), LysM‐Cre‐\ROSA‐mTom/MEGFP+ (middle bar), and LysM‐Cre+\ROSA‐mTom/MEGFP+ (right bar). The graph represents the percent distribution of mTom−/mEGFP‐ (white), mTom+/mEGFP‐ (red), mTom+/mEGFP+ (orange), and mTom−/mEGFP+ (green) in recovered BAL cells in the four quadrants from the flow cytometry analyses from indicated transgenic mice. The data are expressed as means (±SEM). Sample size (*n*) = 3–7/group. anova: ****P < 0.0001.

A previously generated LysM‐Cre+/mTom/mEGFP+ bi‐transgenic line (Saini et al. 2015) was employed to characterize the cell specificity of LysM‐Cre in BAL cells collected from 4 to 5 weeks old mice (Fig. 1; Fig. S2). Flow cytometric evaluation of mTom and mEGFP expressing cells was performed on BAL cells harvested from mice expressing LysM‐Cre alone (to target Cre recombinase expression to myeloid cells under the LysM promoter) (Clausen et al. 1999), ROSA‐mTom/mEGFP alone (the floxed reporter construct ROSA‐mTom/mEGFP) (Muzumdar et al. 2007), or both transgenes (Fig. 1; Fig. S2).

The LysM‐Cre mice, as expected, were null for fluorescent protein expression (Fig. 1, left bar; Fig. S2). The ROSA‐mTom/mEGFP reporter mice exhibited robust ROSA locus expression in BAL cells (96% of which are MΦ, not shown), as indicated by mTom+ expression in ~98% of harvested cells (Fig. 1, middle bar; Fig. S2). Finally, robust LysM‐promoter driven Cre activity in BAL MΦ was evidenced by the presence of ~96% mEGFP+ MΦ in LysM‐Cre+\mTom/mEGFP+ bi‐transgenic mice (Fig. 1, right bar; Fig. S2).

### LysM‐Cre driven DTA expression alters pulmonary MΦ populations

**Figure 2:**
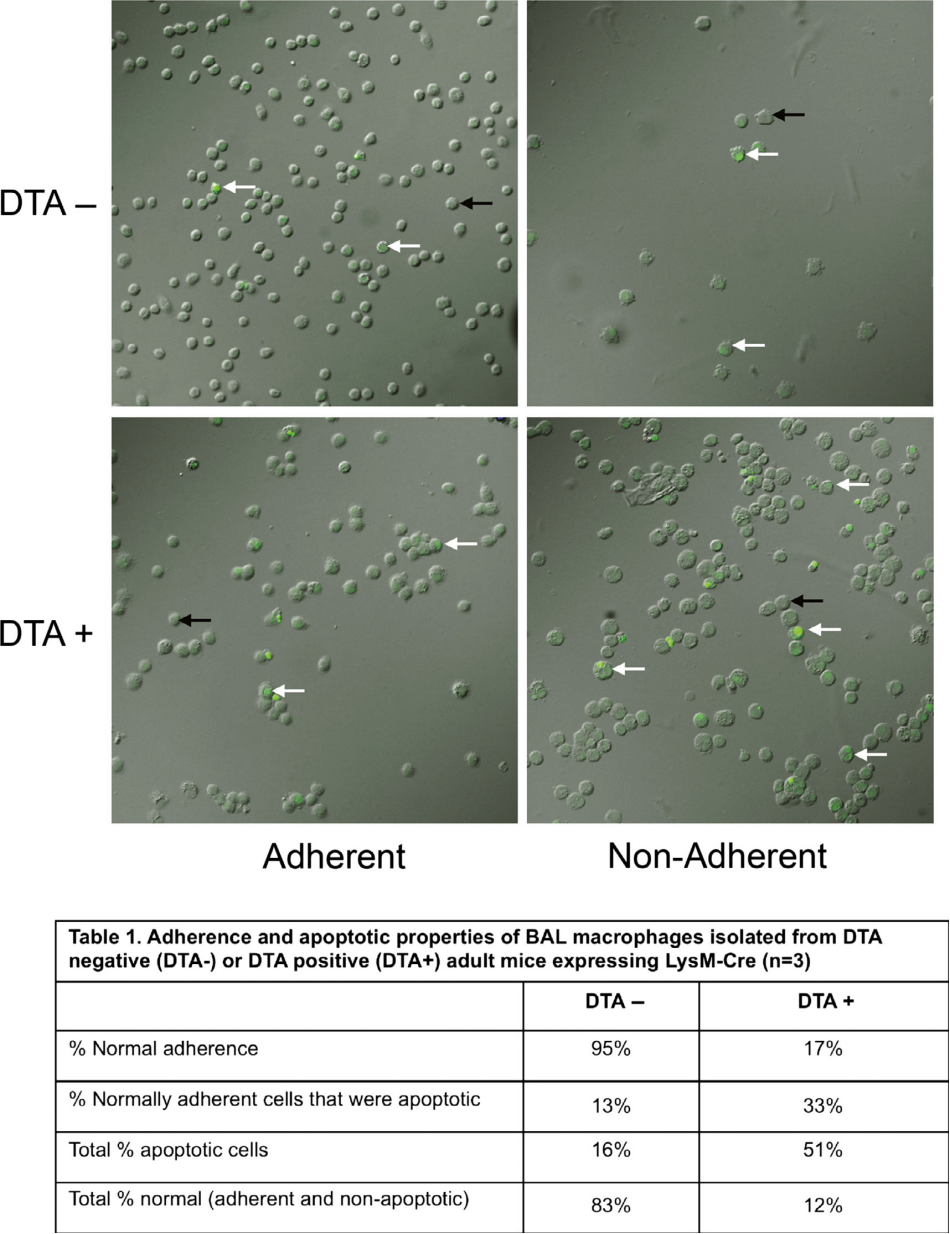
Representative images obtained from the Caspase activity assay as described in the methods and results for both adherent and non‐adherent cells from DTA− and DTA+ mice as indicated. White arrows highlight caspase positive cells, which fluoresce green. Black arrows depict caspase negative cells. Table*:* Adherence and apoptotic properties of BAL MΦ isolated from DTA negative (DTA−) or DTA positive (DTA+) mice expressing LysM‐Cre (*n* = 3).

To evaluate whether DTA expression effectively induced the predicted MΦ apoptosis, a caspase 3/7 apoptosis assay was performed on BAL cells from DTA+ and DTA− mice (Fig. 2). While the original intent was to evaluate only apoptosis via caspase activation, it was noted during the studies that adherence to the assay plate was also altered in harvested MΦ. Thus, two separate phenotypes, adherence and apoptosis, were scored as indexes of MΦ function (Fig. 2).

In MΦ from DTA+ mice, only 17% of MΦ from BAL exhibited normal adherence as compared to 95% from DTA− mice (Fig. 2). The percentage of apoptotic cells was also greater in the DTA+ mice. Cumulatively, only 12% of BAL MΦ from DTA+ mice were classified as “normal” (both functionally adherent and non‐apoptotic) as compared to 83% from DTA− mice. Thus, despite significant numbers of MΦ in the DTA+ mouse lungs, the findings of increased apoptosis and defective adherence suggest large reductions in MΦ function. Attempts to increase the degree of total MΦ depletion by doubling the copy number of DTA alleles by breeding DTA and LysM‐Cre alleles to homozygosity were unsuccessful due to increased neonatal lethality (not shown).

### Effect of DTA expression on BAL MΦ populations

DTA+ (LysM‐Cre+/DTA+/mTom/mEGFP+) and DTA− (LysM‐Cre+/DTA−/mTom/mEGFP+) mice were generated by crossing LysM‐Cre+/mTom/mEGFP and Rosa‐floxed DTA strains. As described previously (Saini et al. 2015), regulation of DTA and mEGFP expression simultaneously by a common ROSA locus facilitated the monitoring of DTA‐induced cell death and reduction in mEGFP+ cells in DTA+ lines.

**Figure 3:**
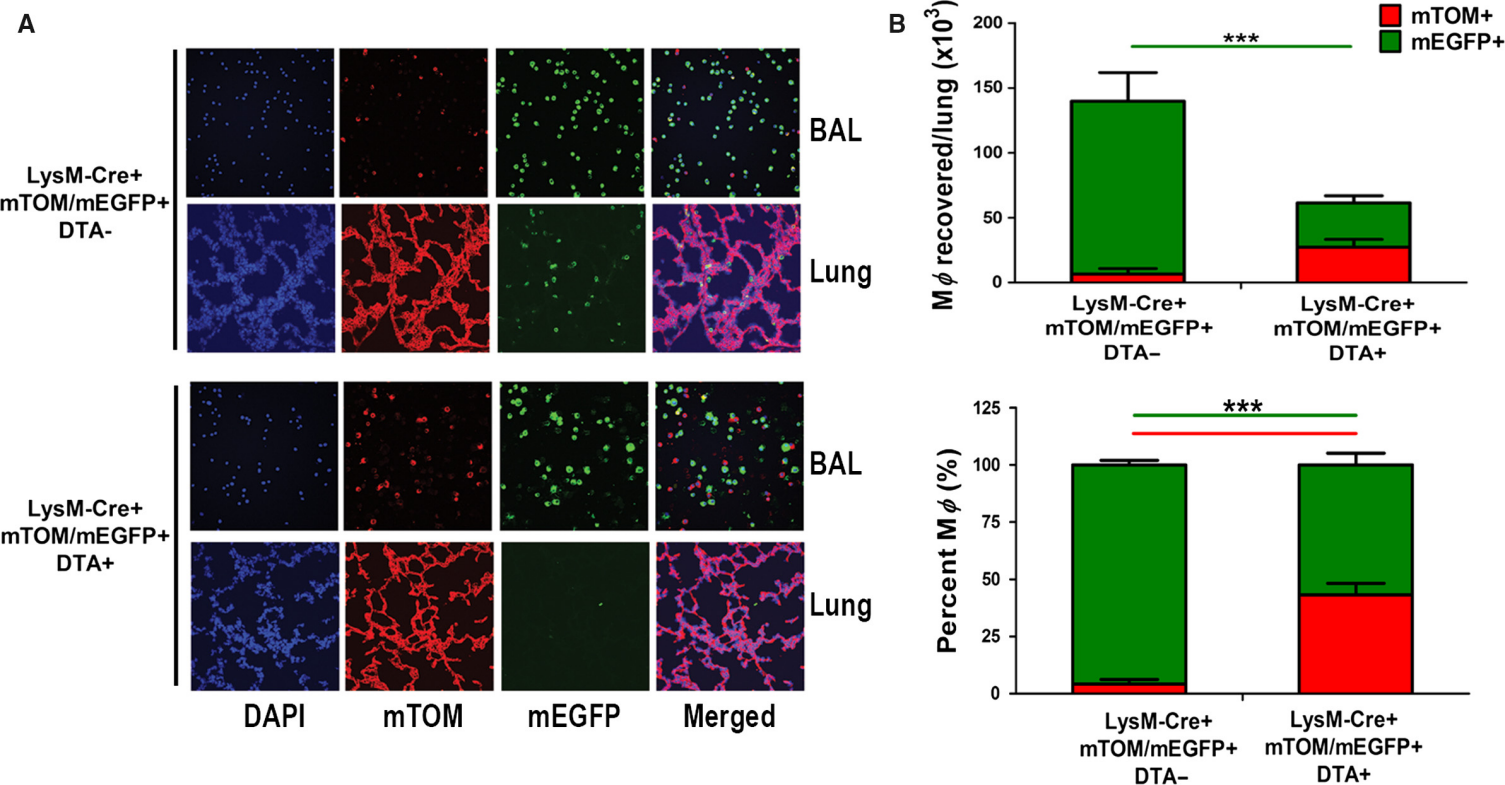
DTA expression targeted to pulmonary MΦ alters the LysM‐expressing cell population. (A) BAL cells and histological lung sections were evaluated for mTom (red; LysM‐Cre negative) or mEGFP (green; LysM‐Cre positive) expression via fluorescence microscopy in DTA− or DTA+ mice. (B) Quantitative assessment of percent cells expressing either mTom and/or mEGFP as determined by counting fluorescent signal on cytospins (*n* = 4). The data are expressed as means (±SEM). anova ***P < 0.001.

DTA+ versus DTA− mice were phenotyped to determine the relative proportions of mTom+ and LysM‐Cre expressing mEGFP+ MΦ (Fig. 3A and B). The absolute MΦ numbers in DTA+ mice were reduced to ~50% compared to DTA− littermates. Interestingly, the absolute numbers of mEGFP+ MΦ were reduced by >75% in DTA+ compared to DTA− mice. The reduction in the absolute MΦ counts in DTA+ mice resulted in the: (Burnett et al. 2004) decreased percentage of mEGFP+ MΦ in DTA+ (54%) versus DTA− mice (~95%) and (Byrne et al. 2015) an increased percentage of mTom+ cells (~46%) compared to DTA− counterparts (~5%) (Fig. 3A and B). In summary, our data suggest that the LysM‐mediated DTA expression produced a BAL cell population depleted in mEGFP+ MΦ but with an increased number of LysM‐ mTom+ MΦ.

**Figure 4:**
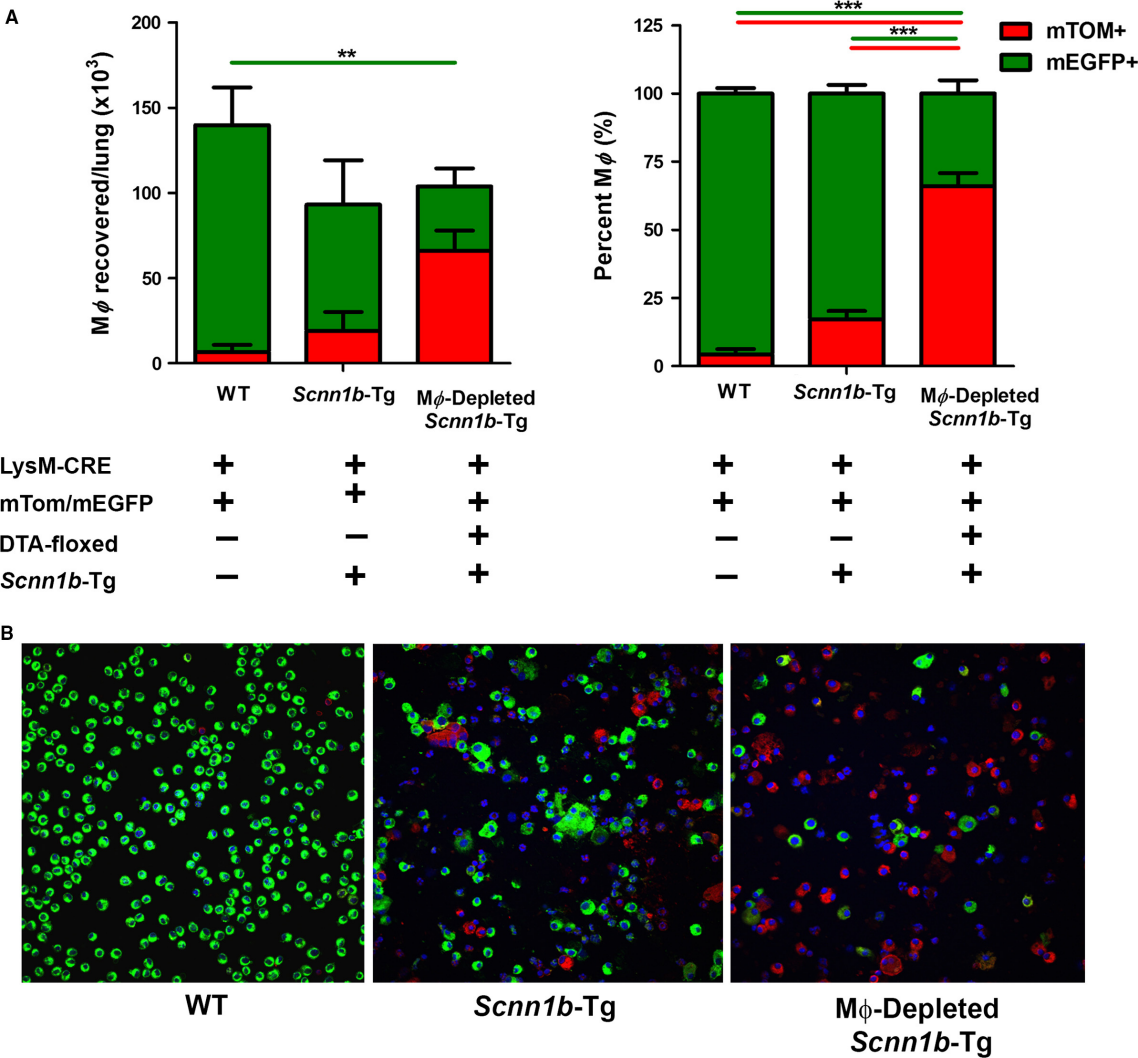
Characteristics of LysM and ROSA promoter activity in *Scnn1b*‐Tg BAL cells. DTA‐expression (LysM‐Cre positive, MΦ‐depleted) leads to loss of mEGFP expression. (A) Histogram indicating the percentage of mTom or mEGFP expressing cells in the presence (+) or absence (−) of the transgenes as indicated (*n *=* *3–5). The data are expressed as means (±SEM). anova **P < 0.01, ***P < 0.001. (B) Representative fluorescent images of BAL cells in LysM‐Cre, reporter positive mice that are either DTA negative (*Scnn1b*‐Tg) or DTA positive (MΦ‐depleted *Scnn1b*‐Tg) mice.

BAL cells recovered from DTA−/WT, DTA−/*Scnn1b*‐Tg and DTA+/*Scnn1b*‐Tg mice were analyzed to determine the relative proportions of mTom+ cells versus mEGFP+ cells. The comparison of DTA−/Scnn1b‐Tg mice to DTA−/WT mice revealed that the absolute number of mEGFP+ MΦ tended to be lower and the absolute number of mTom+ MΦ tended to be higher in DTA−/*Scnn1b*‐Tg mice, resulting in a higher proportion of mTom+ MΦ in DTA−/*Scnn1b*‐Tg (~17%) compared to DTA−/WT mice (~5%) (Fig. 4A and B). DTA transgene expression in *Scnn1b*‐Tg mice resulted in an absolute reduction in mEGFP+ and increase in mTom+ MΦ, producing a larger relative expression of mTom+ MΦ in the DTA+/*Scnn1b*‐Tg (66%) versus DTA−/*Scnn1b*‐Tg 17%) mice (Fig. 4A and B).

### Effect of DTA+ expression on BAL cellularity

**Figure 5:**
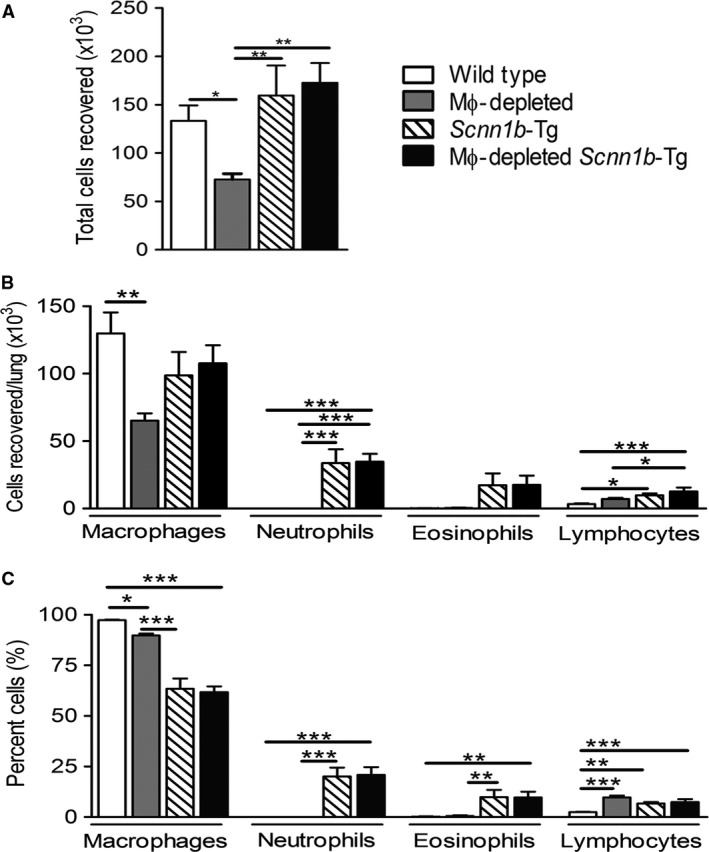
MΦ‐depletion exhibits altered BAL cell composition in WT and *Scnn1b*‐Tg mice. (A) Total and (B) differential cell counts are shown for BAL cells for the genotypes as indicated (*n* = 10–13). (C) Percent compositions of various cell types in the BAL are shown (*n* = 10–13). The data are expressed as means (±SEM). anova *P <* *0.05, **P < 0.01, ***P < 0.001.

The absolute cell numbers in DTA+/WT mice were reduced by 43% which was largely attributable to a ~50% reduction in MΦ counts (Figs. 3 and 5). Importantly, no increase in neutrophil or eosinophils was seen in BALF of DTA+ versus WT mice. As previously reported, DTA−/*Scnn1b*‐Tg mice exhibited only slightly increased cell counts, similar MΦ numbers, but increased neutrophils, eosinophils, and lymphocytes numbers compared to WT mice. However, absolute cell number, MΦ, and neutrophil counts were not different between DTA−/*Scnn1b*‐Tg mice and DTA+/*Scnn1b*‐Tg mice (Fig. 5).

### Effect of MΦ depletion on *Scnn1b*‐Tg lung disease

**Figure 6:**
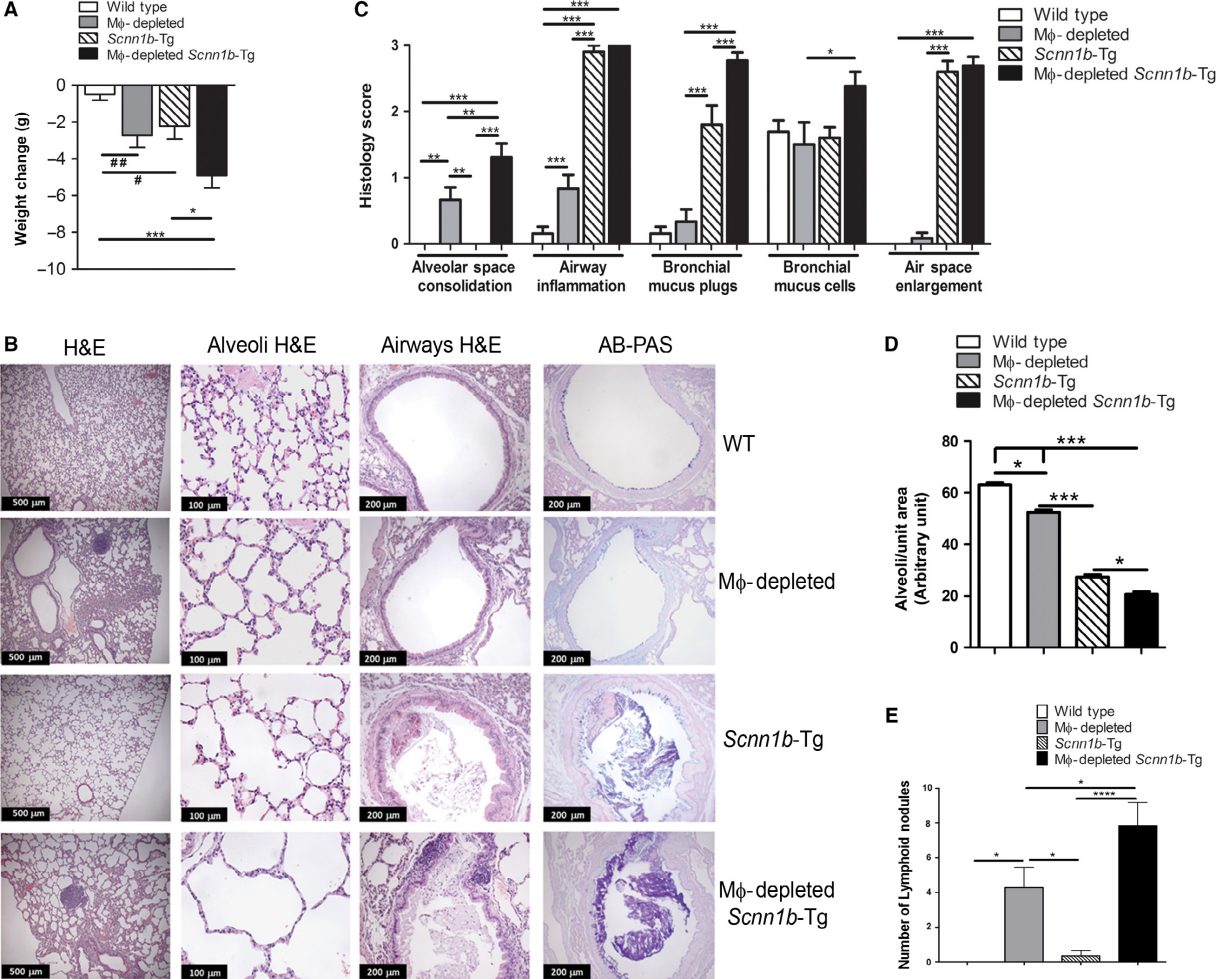
MΦ‐depletion alters the lung pathology in *Scnn1b*‐Tg mice. (A) Body weight change in MΦ‐depleted WT and *Scnn1b*‐Tg mice. The values represent body weight difference between WT and gender‐matched transgenic littermates. The values were generated by subtracting body weight of WT animal from body weight of each gender‐matched transgenic littermate (*n* = 8–12). The data are expressed as means (±SEM). Unpaired Student's *t*‐test ^#^ *P* < 0.05, ^##^ *P* < 0.01; anova *P <* *0.05, ***P < 0.001. (B) MΦ‐depletion produces phenotypes that persist into pre‐adulthood as indicated by representative histological sections for each genotype as indicated. MΦ‐depleted mice exhibit significant pulmonary pathology. (C) Histological scoring for genotypes as indicated is shown (*n* = 10–13). (D) Airspace enlargement was quantified in 4‐ to 5‐week‐old mice using a quantitative method. (Fig. S3) (E) Quantification of alveolar lymphoid nodules in lung sections. The data are expressed as means (±SEM). anova *P < 0.05, **P < 0.01, ***P < 0.001.

DTA+/WT mice did not exhibit gross phenotypic abnormalities except reduced weight compared to gender‐matched DTA−/WT littermates. Reduced weight gain was also observed in DTA−/*Scnn1b*‐Tg mice, and the effects of LysM+ MΦ depletion and *Scnn1b*‐Tg expression appeared to be additive (Fig. 6A).

The DTA+/WT mice exhibited only modest airway inflammation compared to DTA−/WT mice, but did exhibit focal areas of alveolar space consolidation (Fig. 6B). DTA+/*Scnn1b*‐Tg mice exhibited more alveolar consolidation as compared to their DTA−/*Scnn1b*‐Tg littermates. With respect to their respective airways phenotype, airways inflammation was similar in DTA+/*Scnn1b*‐Tg and DTA−/*Scnn1b*‐Tg mice, but mucus plugging and bronchial mucous cells were increased in DTA+/*Scnn1b*‐Tg mice compared to DTA−/*Scnn1b*‐Tg mice (Fig. 6B and C).

Semi‐quantitative histological scoring did not detect significant increases in alveolar space enlargement in (Burnett et al. 2004) DTA+/WT verses DTA−/WT or (Byrne et al. 2015) DTA+/*Scnn1b*‐Tg verses DTA−/*Scnn1b*‐Tg lines (Fig. 6C). However, a significant increase in airspace enlargement was observed with DTA expression in both WT and *Scnn1b*‐Tg mice when alveolar space size was quantified using quantitative morphometric analysis (Fig. 6D; Fig. S3).

**Figure 7:**
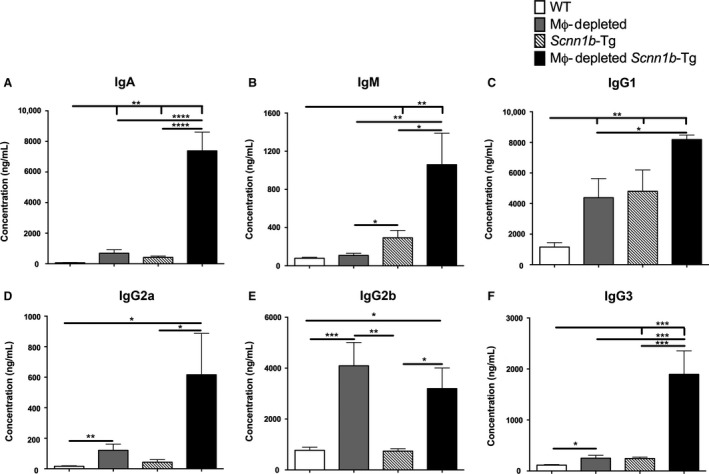
MΦ‐depleted *Scnn1b*‐Tg mice exhibit higher BAL immunoglobulin levels. The BAL concentration (ng/mL) of six major immunoglobulin isotypes (*n* = 5). The data are expressed as means (±SEM). anova *t*‐test^#^ *P < 0.05, **P < 0.01, ***P < 0.001, ****P < 0.0001.

Both DTA+/WT and DTA+/*Scnn1b*‐Tg mice developed lymphoid‐like nodules in the lung parenchyma (Fig. 6E). Some of these nodules were different than lymphoid‐like nodules previously observed in *Scnn1b*‐Tg mice (Livraghi‐Butrico et al. 2012), that is, they tended to be localized away from the conducting airways (Fig. 6B and E). To test whether the lymphoid nodules act as a local source of immunoglobulins, BAL concentrations of six major immunoglobulin isotypes, that is, IgA, IgG1, IgG2a, IgG2b, IgG3, and IgM, were measured (Fig. 7). Except for IgM, all the tested immunoglobulin isotypes were significantly higher in BAL of DTA+/WT mice as compared to DTA−/WT mice. In comparison with DTA−/WT mice, DTA−/*Scnn1b*‐Tg BAL had significantly higher levels of IgA, IgM, IgG1, and IgG3. However, BAL from DTA+/*Scnn1b*‐Tg mice exhibited remarkably higher levels of all the six isotypes. In particular, in comparison with DTA−/*Scnn1b*‐Tg mice, the DTA+/*Scnn1b*‐Tg mice had significantly higher concentration of IgA, IgM, IgG1, IgG2b, and IgG3 (Fig. 7).

The *Scnn1b*‐Tg neonates exhibit microbial infection predominated by bacterial species capable of colonization under microaerophilic conditions in hypoxic airways of these mice (Livraghi‐Butrico et al. 2012). These infections are mostly cleared by the age of 4–5 weeks (Livraghi‐Butrico et al. 2012), due to unknown reasons. We speculated that the compromised MΦ functions in DTA+/*Scnn1b*‐Tg mice might result in defective bacterial clearance. Therefore, using our established methodology (Livraghi‐Butrico et al. 2012; Saini et al. 2015), BAL microbiological analyses were conducted to detect microaerophilic bacterial species in BAL recovered from DTA+/WT, DTA−/*Scnn1b*‐Tg, and DTA+/*Scnn1b*‐Tg mice. Counter‐intuitively, no bacterial colonies were recovered from BAL from DTA+/*Scnn1b*‐Tg or control groups (not shown). The ability of DTA+/*Scnn1b*‐Tg mice to clear neonatal bacterial infections is likely due to the compensatory adaptive responses, that is, appearance of lymphoid nodules (Fig. 6E) and elevated levels of antibacterial immunoglobulins in airspace (Fig. 7).

DTA+/WT mice did not exhibit increased cytokine levels compared to WT controls (Table 1), consistent with an apparent resolution of MΦ apoptosis‐related inflammation with age. The *Scnn1b*‐Tg mice exhibited the pattern of cytokine elevation relative to WT mice as previously reported, for example, marked increase in KC, MIP2, LIX, and MIP1*α*. However, the introduction of LysM+ MΦ depletion onto the *Scnn1b*‐Tg mouse background produced a pattern whereby 14 of the 15 measured cytokines were elevated in DTA+/*Scnn1b*‐Tg versus DTA−/*Scnn1b*‐Tg mice. These data indicated that the mucus‐clearance defect interacted with MΦ‐depletion to increase the concentration of these mediators in the lung (Table 1).

**Table 1:** Cytokine responses across designated groups

Cytokine (pg/mL)	WT	MΦ‐depleted WT	*Scnn1b*‐Tg	MΦ‐depleted *Scnn1b*‐Tg	Lower limits of detection
KC	9.0 ± 0.2*	23.9 ± 3.8	299.2 ± 37.7# ^,^ *	597.2 ± 70.8#	3.1
MIP2	2.7 ± 0.1*	2.7 ± 0.03	30.0 ± 8.2# ^,^ *	117.3 ± 8.3#	3.2
MIP1*α*	7.4 ± 0.7#	11.1 ± 2.8	33.5 ± 4.3#	31.9 ± 1.8	4.1
LIX	22.6 ± 0.0#	22.6 ± 0.0	923.4 ± 158.9#	2325.0 ± 626.6	22.6
TNF*α*	0.6 ± 0.0	0.6 ± 0.0	2.5 ± 0.6*	3.7 ± 0.8*	3.2
IL5	1.1 ± 0.6#	1.0 ± 0.3	19.2 ± 4.1#	83.2 ± 26.9	2.9
IL10	5.7 ± 1.7#	6.3 ± 0.9	1.2 ± 0.3#	1.3 ± 0.4	3.2
M‐CSF	0.1 ± 0.0	0.1 ± 0.0	0.2 ± 0.1*	2.6 ± 0.9*	2.9
IL17	0.0 ± 0.0	0.0 ± 0.0	0.0 ± 0.0*	2.2 ± 0.9*	3.1
IL6	6.0 ± 0.0	6.0 ± 0.0	11.2 ± 4.3	15.9 ± 5.6	3.7
IP10	5.8 ± 0.1	11.5 ± 1.9	11.9 ± 1.6	24.5 ± 6.5	3.2
MCP1	4.0 ± 1.9	3.6 ± 1.0	13.4 ± 7.9	15.1 ± 4.9	20.3
IL4	0.4 ± 0.3	0.2 ± 0.0	3.9 ± 1.7	11.6 ± 6.5	3.2
Eotaxin	1.2 ± 0.5	3.3 ± 1.7	4.2 ± 1.4	8.6 ± 3.4	2.7
IL1*β*	0.8 ± 0.0	2.4 ± 1.4	0.9 ± 0.1	3.1 ± 1.5	2.5

Cytokine Levels (pg/mL) in BAL. Values bearing identical designations (^#^ or *) within a row represent significant difference. Yellow Background: Significantly higher compared to macrophage‐depleted WT group. Green Background: Significantly higher compared to macrophage‐depleted *Scnn1b*‐Tg group. The standard errors of the mean (±SEM) values for some cytokines were 0.0 because these cytokine levels were below detection limits and mean were assigned the values equal to the lowest assay detection limit. Values less than the lower limit of detections were obtained by extrapolation. *N *=* *5–6, *P *<* *0.05.

## Discussion

Given their remarkable plasticity (Byrne et al. 2015), MΦ possess the capability of sensing and responding to inflammatory triggers in distressed airspaces in pulmonary diseases and, accordingly, influence the inflammatory responses. MΦ responses *per se* and MΦ‐epithelial interactions, however, are poorly understood, thus in vivo investigations in suitable airway disease models are warranted. One relevant model of human CB, that is, the *Scnn1b*‐Tg mouse, provides a suitable tool to understand the MΦ‐mediated responses in the muco‐obstructive airspaces.

To understand the roles of MΦ in the muco‐obstructive lung disease in *Scnn1b*‐Tg mice, we conducted a study of neonatal *Scnn1b*‐Tg with and without DTA‐mediated MΦ depletion (Saini et al. 2015). The study revealed several important findings. First, the MΦ depletion strategy was successful in the elimination of almost the entire LysM‐Cre+ MΦ population, however, the compensatory recruitment of immature MΦ (LysM‐Cre‐) resulted in no net change in the absolute MΦ counts in DTA+ neonates compared to DTA− neonates. Second, MΦ depletion led to persistent alveolar infection in ~25% of pups, suggesting absolute requirement of MΦ in eliminating alveolar space bacterial infections. Third, MΦ depletion induced more pronounced neutrophil recruitment in DTA+ mice with or without *Scnn1b‐*Tg background, likely reflecting a response to signals released by DTA‐induced MΦ depletion. Fourth, superimposition of MΦ depletion on the airway mucus clearance defect of *Scnn1b‐*Tg resulted in alveolar infection in a greater proportion of DTA+/*Scnn1b‐*Tg pups (~51%) suggesting an interaction between regional host defense systems, that is, mucociliary clearance (MCC) and alveolar MΦ. Collectively, this study revealed critical role of MΦ in the clearance of neonatal bacterial infection, in synergism with the MCC system. Whether similar interactions exist between MΦ and MCC in older mice remained unexplored.

Therefore, we focused our investigations on MΦ‐MCC interactions in 4‐ to 5‐week‐old *Scnn1b‐*Tg mice. It is noteworthy that the DTA+ mice used in this study were survivors from the litters that had early postnatal mortalities due to emaciation associated with lethal pneumonia in MΦ depleted neonates. The study was focused on assessing the effect of MΦ depletion on CB disease‐relevant endpoints, including bacterial clearance, mucus plugging and mucous cell metaplasia, airway inflammation, BAL cellular disturbances, and airspace enlargement.

The first experiments assessed the phenotype of BAL MΦ from DTA+ versus DTA− mice. The experiments were designed to allow comparison of phenotype between previously published neonates (Saini et al. 2015) and 4‐ to 5‐week‐old mice in this study. In contrast to DTA− neonates, in which only ~62% of MΦ were mature as indexed by monitoring LysM‐Cre activity, that is, mEGFP expression (Saini et al. 2015), >96% of MΦ harvested from 4‐ to 5‐week‐old WT mice were mature (LysM‐Cre+, i.e., mEGFP+) (Fig. 3). Also, in contrast to DTA+ neonates, in which LysM‐Cre mediated DTA expression did not reduce absolute number of BAL MΦ (Saini et al. 2015), 4‐ to 5‐week‐old DTA+ mice exhibited significant reductions in MΦ numbers to ~50% of WT levels. The MΦ population recovered from 4‐ to 5‐week‐old DTA+ mice was equally divided between mature (LysM‐Cre expressing) and immature (non‐expressing) cell‐types, with a majority of both being functionally defective (either apoptotic or non‐adherent). The sustenance of ~50% total MΦ likely reflects the time lag between recruitment of immature MΦ and LysM‐Cre mediated induction DTA expression and, in turn, apoptotic death. The age‐dependent differences in MΦ populations are consistent with variations in the proportions of embryonic‐derived MΦ in neonates versus older mice (Guilliams et al. 2013), and/or differences in MΦ recruitment/proliferation rates at the two ages. Based on the data obtained from 4‐ to 5‐week‐old mice, the description of this model of MΦ depletion as “a model of MΦ‐depletion coupled to variable recruitment of LysM‐ negative, functionally defective MΦ or monocytes” is still applicable (Saini et al. 2015).

The BAL neutrophilia associated with MΦ depletion noted in DTA+/WT neonates (Saini et al. 2015) and other MΦ‐depletion models [MAFIA mice, a different model of MΦ depletion (Burnett et al. 2004), and clodronate liposome‐induced MΦ depletion (Nakamura et al. 2005) was absent in older DTA+ mice. Except KC, the neutrophil chemokines, including LIX and MIP2, were not significantly different in BALF from DTA+/WT and DTA−/WT mice (Table 1). We speculate that the effect of MΦ depletion in neonatal lungs results in compensatory increase in neutrophil recruitment that is essential for the clearance of apoptotic cells. In contrast, the older mice with developed lungs with minimal apoptotic cells do not require significant macrophage/neutrophil mediated clearance of apoptotic debris. Although the levels of neutrophil chemokines were significantly elevated in BALF from DTA−/Scnn1b‐Tg versus DTA− (or DTA+) mice, the expected increase in neutrophilic recruitment was not observed in DTA+/Scnn1b‐Tg. Since LysM‐Cre is known to target neutrophils (Clausen et al. 1999), there exist a possibility of numerical depletion of neutrophils, in blood stream or in airspaces, which compromises the neutrophil recruitment rate.

The bacterial burden consistently present in *Scnn1b‐*Tg neonates is diminished by the age of 4–5 weeks which most likely suggests improved phagocytic and bactericidal capabilities of phagocytes due to age‐dependent maturation of the innate and adaptive immune system. To determine whether numerical deficiency of MΦ would compromise improved bacterial clearance seen in DTA−/Scnn1b‐Tg mice, we micro‐aerobically cultured BAL from the lungs of older mice with different genotypes (Saini et al. 2015). Surprisingly, the BAL microbiological analyses revealed an absence of bacterial infection in all genotypes. We speculated that the age‐dependent clearance of bacterial infection, despite numerical and functional MΦ deficiencies, reflects the maturation of the adaptive immune response. To test this notion, we quantitated alveolar lymphoid nodules in lung sections (Moyron‐Quiroz et al. 2004) and analyzed immunoglobulin levels in BAL (Daniele 1990). The alveolar lymphoid nodules, which are normally absent in WT mice at 4–5 weeks of age, became prominent in DTA+/WT, and they were more prevalent in *Scnn1b*‐Tg mice with DTA+ expression. The appearance of similar lymphoid tissues has been reported in Myd88^−/−^ *Scnn1b*‐Tg mice but only at 8 weeks of age (Livraghi‐Butrico et al. 2012). This suggests that the macrophage deficiency in DTA+ mice compromises multiple pathways of host defense (Roy et al. 2014) including Myd88‐mediated, thus triggering early appearance of lymphoid tissues. Furthermore, immunoglobulin isotyping in BAL revealed significantly increased levels of immunoglobulins in DTA+/*Scnn1b*‐Tg mice. Collectively, these data suggest that an amplified adaptive response prevented bacterial infection in DTA+/Scnn1b‐Tg mice. Indeed, this development of robust adaptive immune responses may be the reason why the alveolar pneumonia was restricted to the neonatal period.

With respect to protective versus adverse effects of persistent MΦ activation on CB severity, our data suggest that MΦ contribute to contain disease severity in the *Scnn1b*‐Tg mouse. Notably, DTA+/Scnn1b‐Tg mice exhibited increased mucus plugging, lymphocytic accumulation, and increased cytokine levels compared to DTA−/Scnn1b‐Tg mice. These data suggest that loss of airway MΦ‐derived signals that normally dampen mucin secretion and inflammation may contribute to worsening of muco‐obstructive disease. Similarly, partial numerical deficiency of MΦ may compromise clearance of mucus‐trapped biotic and abiotic proinflammatory materials, perpetuating the inflammatory response.

These findings, with respect to mucus load and MΦ numbers, contrast with recent findings in models of Type 2 (Th2) allergic lung inflammation (Lee et al. 2014; Zaslona et al. 2014; Mathie et al. 2015). In these models, MΦ‐depletion attenuated, rather than enhanced, mucous cell metaplasia, and other Th2 responses in older mice. These contrasting results highlight the importance of considering the role of MΦ's within the context of the specific disease model being studied. Collectively, these findings suggest that normal MΦ signaling amplifies allergic mucus responses but attenuates similar responses in the context of infectious muco‐obstructive lung disease. In other words, the development of therapies directed at MΦ function might require tailor‐made approaches for individual disease type.

Morphological assessment of lung sections revealed interesting association between DTA+ expression and increased alveolar‐space enlargement. Since MΦ‐specific MMP12 expression has been associated with alveolar‐space enlargement phenotype in *Scnn1b*‐Tg mice (Hautamaki et al. 1997), it was surprising to observe alveolar‐space enlargement in DTA+/WT mice. Although still unclear, we speculate the contribution of neutrophil‐specific proteases in tissue remodeling during early postnatal lung development that manifests as the alveolar‐space enlargement in older mice (Greene and McElvaney 2009). Overall, it appears that alterations in BAL cell composition may contribute to the protease:antiprotease imbalance, which is important for alveolar wall development, but the cause‐effect relationship requires further study.

One caveat to our approach of macrophage depletion is that, due to the unavailability of a cre expressing strain that uses exclusively macrophage‐specific promoter, we selected LysM‐Cre strain that, although capable of targeting a majority of airspace MΦ, is known to target other myeloid cell populations, including neutrophils (Clausen et al. 1999) and ~45% of classical dendritic cells (cDC) (McCubbrey et al. 2017). Therefore, there exists a possibility that the observed phenotype in DTA+/Scnn1b‐Tg mice may reflect a response due to depletion of multiple cell types, including MΦ, neutrophils, and cDC.

In conclusion, this study provides several insights into the role of MΦ in CB. First, 4‐ to 5‐week‐old mice exhibited robust activity of LysM promoter in BAL MΦ that, when used to induce DTA expression, resulted in ~50% depletion of MΦ. The residual MΦ population likely reflected the time lag between continuously recruited immature MΦ into the lung and LysM‐Cre mediated apoptosis. Second, superimposition of MΦ depletion on MCC defect worsened mucous cell metaplasia and airway mucus plugging in *Scnn1b*‐Tg mice. Third, surprisingly, the MΦ depletion did not increase the likelihood of bacterial infection, suggesting a robust upregulation of host immune responses, for example, immunoglobulins. Fourth, MΦ depletion resulted in increased alveolar space enlargement in *Scnn1b*‐Tg mice, possibly reflecting age‐dependent protease:antiprotease imbalance mediated by neutrophils. The present model may be used to investigate mechanisms regulating MΦ recruitment and MΦ‐epithelial interactions in diseased airspace compartments.

## Conflict of Interest

None declared.

## Data Accessibility

## Supporting information




**Figure S1.** Description of transgenic mice used for breeding. LysM‐Cre strain uses endogenous Lysozyme M (LysM) promoter to control the expression of Cre recombinase (*Cre*) transgene. The Rosa‐(mTom−PolyA)^Fx/Fx^/mEGFP reporter strain expresses mTom (or mEGFP) fluorescent protein in the absence (or presence) of Cre recombinase, respectively. The Rosa‐(NeoR‐PolyA)^Fx/Fx^/DTA reporter strain expresses DTA protein in the presence of Cre recombinase. The *Scnn1b*‐Tg strain overexpresses the *Scnn1b* transgene in the club cells via the rat CCSP (*Scgb1a1*) promoter.

**Figure S2** ***.*** Flow cytometry for BAL cells harvested from LysM‐Cre+\ROSA‐mTom/mEGFP‐ (Left panel), LysM‐Cre‐\ROSA‐mTom/mEGFP+ (middle panel), and LysM‐Cre+\ROSA‐mTom/mEGFP+ (right panel). For all figures, the *Y*‐axis is measuring mTom (red) and the *X*‐axis mEGFP (green). Genotypes are indicated below the flow cytometry output. Histograms indicate the percent of cells showing fluorescence as indicated.

**Figure S3.** Morphometric analysis of alveolar topology. Representative raw (left) and analyzed photographs (right) depicting methodology used to determine alterations in alveolar topology. Each individual red line (right column) represents an alveolus. The total number of red lines was calculated in all the 12 images and data was analyzed as described in methods.
