# Supplementary figures and images for: Effect of LysM+ macrophage depletion on lung pathology in mice with chronic bronchitis
Source: Physiol Rep. 2018 Apr 18;6(8):e13677. doi: 10.14814/phy2.13677 (PMC5904692; doi:10.14814/phy2.13677)

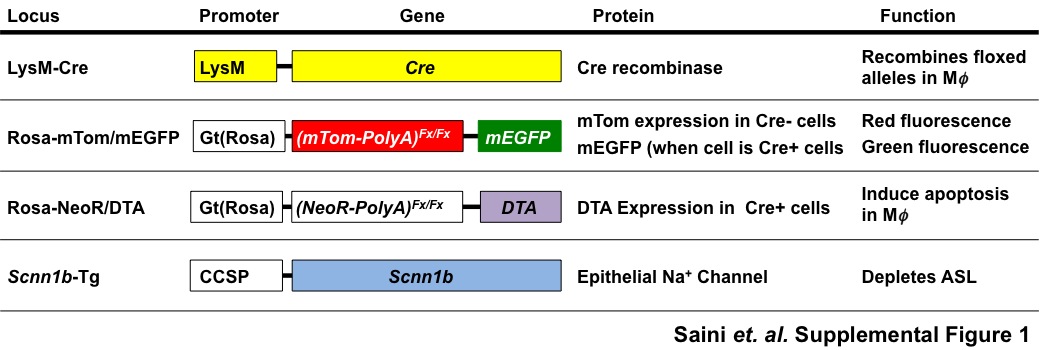

Supplement: Supplementary file 1 — Figure S1. Description of transgenic mice used for breeding. LysM‐Cre strain uses endogenous Lysozyme M (LysM) promoter to control the expression of Cre recombinase (Cre) transgene. The Rosa‐(mTom−PolyA)Fx/Fx/mEGFP reporter strain expresses mTom (or mEGFP) fluorescent protein in the absence (or presence) of Cre recombinase, respectively. The Rosa‐(NeoR‐PolyA)Fx/Fx/DTA reporter strain expresses DTA protein in the presence of Cre recombinase. The Scnn1b‐Tg strain overexpresses the Scnn1b transgene in the club cells via the rat CCSP (Scgb1a1) promoter. [file PHY2-6-e13677-s001.jpg]

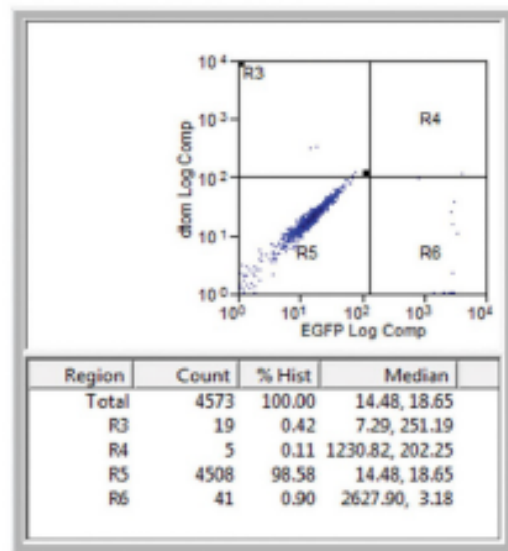

LysM-Cre  
mTOM/mEGFP

+

---

-

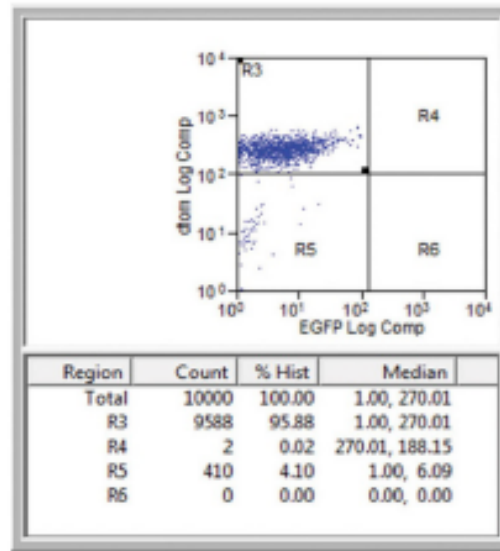

-

---

+

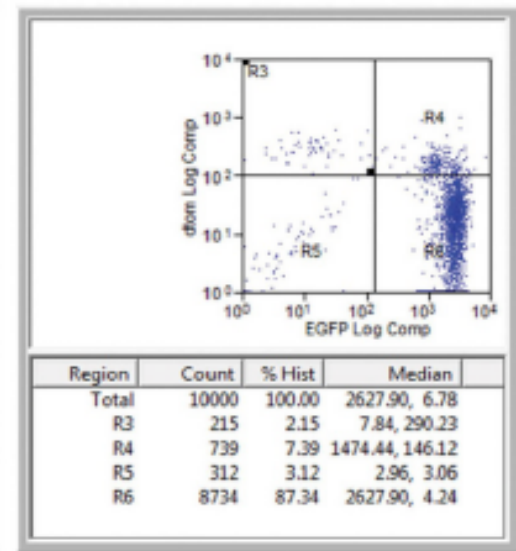

+

---

+

Saini *et. al.*, Supplemental Figure 2

Supplement: Supplementary file 2 — Figure S2 . Flow cytometry for BAL cells harvested from LysM‐Cre+\ROSA‐mTom/mEGFP‐ (Left panel), LysM‐Cre‐\ROSA‐mTom/mEGFP+ (middle panel), and LysM‐Cre+\ROSA‐mTom/mEGFP+ (right panel). For all figures, the Y‐axis is measuring mTom (red) and the X‐axis mEGFP (green). Genotypes are indicated below the flow cytometry output. Histograms indicate the percent of cells showing fluorescence as indicated. [file PHY2-6-e13677-s002.pdf]

Wild Type

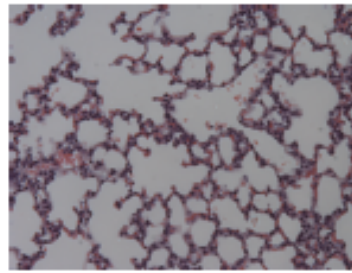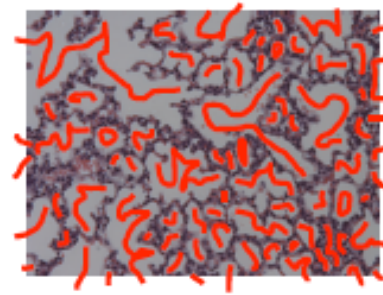

MΦ-Depleted

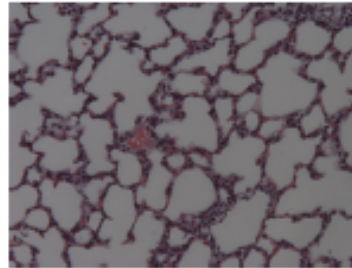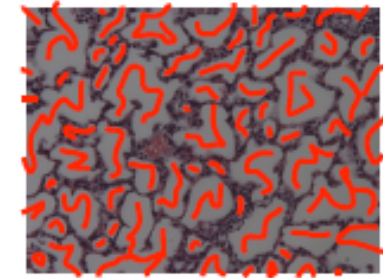

*Scnn1b*-Tg

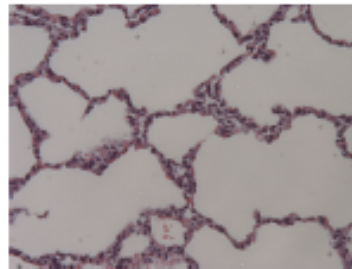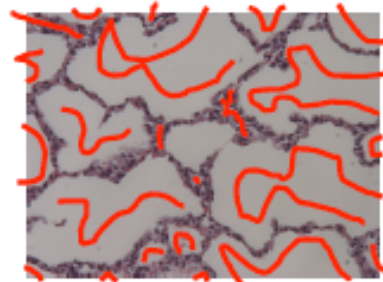

MΦ-Depleted *Scnn1b*-Tg

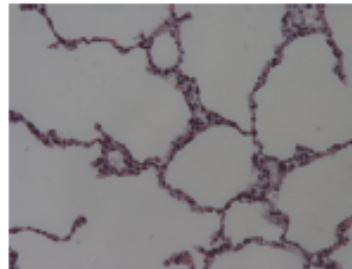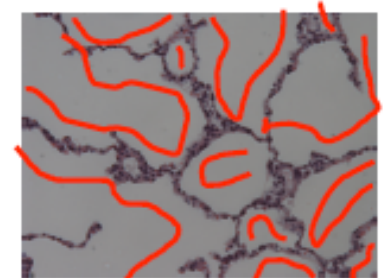

Raw Images

Analyzed Images

Supplement: Supplementary file 3 — Figure S3. Morphometric analysis of alveolar topology. Representative raw (left) and analyzed photographs (right) depicting methodology used to determine alterations in alveolar topology. Each individual red line (right column) represents an alveolus. The total number of red lines was calculated in all the 12 images and data was analyzed as described in methods. [file PHY2-6-e13677-s003.pdf]
